# Point-of-care ultrasound for the evaluation of dental abscesses in the pediatric emergency department

**DOI:** 10.1007/s43678-026-01101-w

**Published:** 2026-02-26

**Authors:** Itai Gross, Maria Nassar, Noa Guzner, Imri Granot, Naama Pines, Lea Ohana Sarna Cahan, Yshia Langer, Saar Hashavya

**Affiliations:** 1 Department of Pediatric Emergency Medicine, Hadassah Medical Center, Ein Kerem, Kiryat Hadassah, POB 12000 Jerusalem, Israel; 2 Department of Pediatric Intensive Care, Hadassah Medical Center, Jerusalem, Israel; 3 Faculty of Dental Medicine, Department of Oral and Maxillofacial Surgery, Hadassah and Hebrew University Medical Center, Jerusalem, Israel

**Keywords:** Dental abscess, Point-of-care ultrasound, Pediatric emergency department, Abcès dentaire, Échographie au point d’intervention, Service d’urgence pédiatrique

## Abstract

**Background:**

Facial cellulitis is a common presentation at pediatric emergency departments. Prompt diagnosis of dentoalveolar abscesses is crucial for initiating appropriate treatment and minimizing morbidity. While imaging modalities such as computed tomography (CT) and a panoramic X*-*ray are available, they are not often used for pediatric patients given the known risks of exposure to radiation and the need for sedation.

**Methods:**

We conducted a prospective diagnostic accuracy study at a tertiary pediatric emergency department to evaluate point-of-care ultrasound (POCUS, index test) for diagnosing dentoalveolar abscesses in children aged 0–18 years. The reference standard was surgical drainage findings by a maxillofacial surgeon. Pediatric emergency medicine physicians performed soft tissue facial ultrasounds following brief standardized training. Diagnostic accuracy was assessed by calculating sensitivity, specificity, positive predictive value, and negative predictive value. Abscess volume was calculated using the ellipsoid formula.

**Results:**

Thirty-three patients were recruited. POCUS demonstrated a sensitivity of 100% (95% CI 84.6–100), specificity of 64% (95% CI 30.8–89.1), positive predictive value of 85% (95% CI 65.1–95.6), and negative predictive value of 100% (95% CI 59.0–100).The abscess volume, as calculated using ultrasound, ranged from 0.004 to 2.16 cm^3^.

**Conclusion:**

These findings demonstrate that point-of-care ultrasound is a valuable diagnostic tool for frontline emergency physicians, allowing accurate bedside identification of dentoalveolar abscesses and guiding timely management while minimizing unnecessary imaging and procedures.

## Résumé

**Contexte:**

La cellulite faciale est une présentation courante dans les services d’urgence pédiatrique. Un diagnostic rapide des abcès dento-alvéolaires est crucial pour initier un traitement approprié et minimiser la morbidité. Bien que des modalités d’imagerie telles que la tomodensitométrie (TDM) et une radiographie panoramique soient disponibles, elles ne sont pas souvent utilisées pour les patients pédiatriques compte tenu des risques connus d’exposition aux radiations et du besoin de sédation.

**Méthodes:**

Nous avons mené une étude prospective sur la précision du diagnostic dans un service d’urgence pédiatrique tertiaire pour évaluer l’échographie au point de soins (POCUS, test d’indice) pour diagnostiquer les abcès dento-alvéolaires chez les enfants âgés de 0 à 18 ans. La norme de référence était les résultats du drainage chirurgical par un chirurgien maxillo-facial. Les médecins en médecine d’urgence pédiatrique ont effectué des échographies faciales des tissus mous après une brève formation standardisée. La précision diagnostique a été évaluée en calculant la sensibilité, la spécificité, la valeur prédictive positive et la valeur prédictive négative. Le volume de l’abcès a été calculé en utilisant la formule ellipsoïde.

**Résultats:**

Trente trois patients ont été recrutés. POCUS a démontré une sensibilité de 100 % (IC à 95 % 84,6–100), une spécificité de 64 % (IC à 95 % 30,8–89,1), une valeur prédictive positive de 85 % (IC à 95 % 65,1–95,6) et une valeur prédictive négative de 100 % (IC à 95 % 59,0–100). Le volume de l’abcès, calculé à l’aide d’une échographie, variait de 0,004 à 2,16 cm3.

**Conclusion:**

Ces résultats démontrent que l’échographie au point de service est un outil de diagnostic précieux pour les médecins d’urgence de première ligne, permettant une identification précise au chevet des abcès dento-alvéolaires et guidant la prise en charge en temps opportun tout en minimisant les examens d’imagerie et procédures inutiles.

## Introduction

Facial cellulitis is a common reason for pediatric emergency visits and may have odontogenic or non-odontogenic causes, with roughly half of the cases attributed to odontogenic infection [1]. In children, the most frequent odontogenic source is periapical infection, where bacterial invasion of the root canal leads to soft tissue inflammation and sometimes dentoalveolar abscess formation [2] Timely diagnosis is critical, as management hinges on early drainage and extraction, which shorten illness duration and reduce morbidity [3]. Although CT and panoramic radiographs are often used in adults, their role in children is limited by radiation exposure and the frequent need for sedation [4, 5]. Ultrasonography of the dentoalveolar ridge has shown promise in adults [6], yet pediatric use remains less explored [6, 7]. To address these limitations, this study prospectively evaluated the diagnostic accuracy of point-of-care ultrasound (POCUS) for detecting dentoalveolar abscesses in children and adolescents, using surgical drainage findings as the reference standard.

## Methods

### Design and setting

This prospective study was conducted at Hadassah Medical Center, a tertiary hospital in Jerusalem, in the pediatric emergency department, which receives on average 24,000 pediatric visits annually.

### Participants

All individuals aged 0–18 years, who presented to the pediatric emergency department with a suspected dental abscess, underwent evaluation by a maxillofacial surgeon. In cases where the decision was made to drain the abscess, the parents or guardians were asked whether they would be willing to have their child undergo a soft tissue facial POCUS performed by the pediatric emergency medicine physician to assess the abscess size. Informed consent was obtained from all families. The POCUS examination did not influence the decision to drain the abscess.

### Index test (POCUS)

Bedside POCUS was conducted using either the SonoSite M-turbo (Bothell, WA) with a linear transducer of 6–13 MHz or the Mindray MX7 (Mindray Bio-Medical Electronics Co., Ltd.) with a linear transducer of 6–14 MHz. Suspected areas were scanned in two orthogonal planes, with a comparison to the contralateral side. Any abnormal fluid collection was measured, and then each ultrasound examination was documented and saved to the ultrasound hard drive.

**Fig. 1:**
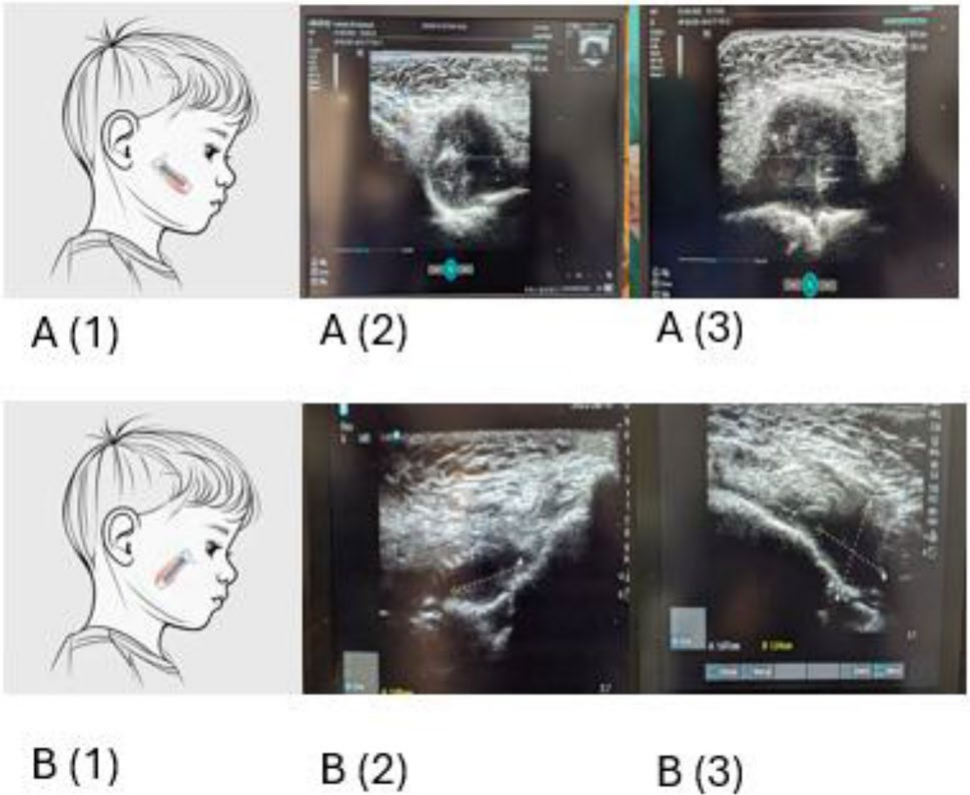
**A** Long-axis illustration of transducer (1) and ultrasound image of a dentoalveolar abscess with size measurement (2), (3). **B** Short-axis illustration of transducer (1) and ultrasound image of a dentoalveolar abscess with size measurement (2), (3)

Four pediatric emergency medicine physicians (two fellows and two consultants) performed all scans. Each had completed a 2 day pediatric POCUS course and had more than 4 years of clinical POCUS experience in the pediatric emergency medicine. None of the operators had completed a formal POCUS fellowship. To standardize acquisition, the first two scans performed by each operator were directly supervised by the POCUS instructor; all subsequent scans were performed independently. The transducer placement for facial swelling imaging is illustrated in the drawing in Fig. 1.

### Outcomes

After the POCUS scan but prior to drainage, the scanning pediatric emergency medicine physician recorded lesion dimensions and calculated volume, then made a binary prediction (“significant pus present” versus “no significant pus”) based on typical sonographic features of abscesses. No fixed volume cutoff was prespecified for the prediction; exploratory volume thresholds were evaluated post hoc. All abscesses were drained via open surgical procedure performed by the maxillofacial surgeon. Fluid volume was not aspirated or formally measured. The maxillofacial surgeon, who was blind to the pediatric emergency medicine physician predication, judged whether a ‘significant’ amount of pus was present, defined as a collection that in their clinical judgment required surgical drainage to achieve resolution. Abscess volume was determined using the ellipsoid formula: 4/3 π (rx) (ry) (rz).

### Statistical analysis

Continuous variables were summarized as mean (standard deviation) and categorical variables as counts (percent). Diagnostic accuracy of POCUS for predicting clinically significant pus at drainage was assessed using sensitivity, specificity, positive predictive value, negative predictive value, and likelihood ratios (LR + and LR −). Exact 95% binomial confidence intervals were calculated for sensitivity and specificity. Confidence intervals for likelihood ratios were calculated using the log method; when a contingency table cell contained zero, the Haldane–Anscombe correction (adding 0.5 to each cell) was applied to allow interval estimation.

Exploratory analyses were performed to examine the diagnostic accuracy of ultrasound-derived abscess volume thresholds. These thresholds were not prespecified and should be considered post hoc.

## Results

Of the 39 patients who presented with dental abscesses, a total of 33 patients (85%) were successfully recruited over the 3-month study period. The average age of the patients was 7.5 years (SD ± 3) and ranged from 3 to 14 years. Of these patients, 52% were male. The average volume of the abscesses, as calculated using the ellipsoid formula, was 1.1 cm^3^ (95% CI 0.7, 1.6 cm^3^).

Based on the ultrasound prediction, 26 of 33 patients were judged to have significant pus by the pediatric emergency medicine physicians, and 22 were confirmed at drainage (true positives). In all seven cases where ultrasound predicted no pus, drainage confirmed none (true negatives). The resulting diagnostic accuracy measures were: sensitivity 100% (95% CI 85–100), specificity 64% (95% CI 31–89), positive likelihood ratio 2.75 (95% CI 1.26–6.01), and negative likelihood ratio 0.00 (95% CI 0.00–0.56, estimated using the Haldane–Anscombe correction). The positive predictive value was 85% (95% CI 65.1–95.6), and a negative predictive value of 100% (95% CI 59.0–100) (Table 1).

**Table 1:** Breakdown of the predictions and outcomes based on the ultrasound scans

	Drainage +	Drainage −	Total
POCUS +	22 (true positive)	4 (false positive)	26
POCUS −	0 (false positive)	7 (true negative)	7
Total	22	11	33

When applying a calculated abscess volume of more than 0.03 cm^3^ as indicative of significance, the POCUS predictions exhibited similar characteristics, with a sensitivity, specificity, positive predictive value, and negative predictive value all consistent at 100%, 64%, 85%, and 100%, respectively. Raising the cutoff to 0.2 cm^3^ resulted in a sensitivity of 86%, a specificity of 91%, a positive predictive value of 95%, and a negative predictive value of 77%.

## Discussion

### Interpretation of findings

Our results affirm the potential diagnostic utility of point-of-care ultrasound (POCUS) as a clinically effective adjunct for the evaluation of pediatric dentoalveolar abscesses. Following brief, standardized training, pediatric emergency medicine physicians demonstrated high diagnostic accuracy in distinguishing between cases necessitating surgical drainage and those not warranting intervention. The observed high negative predictive value is particularly salient, suggesting that a negative POCUS examination may reliably exclude clinically significant abscess formation, thereby minimizing unnecessary surgical procedures. But the wide confidence intervals and the possibility of spectrum bias necessitate cautious interpretation.

### Comparison to previous studies

Adult studies of POCUS for odontogenic and soft tissue infections have yielded mixed results, with some reporting high specificity and others more modest performance. While pediatric literature on POCUS for skin and soft tissue infections remains less extensive than in adults, it is steadily growing, indicating that even clinicians with limited training may achieve clinically useful accuracy. Our prospective pediatric ED findings contribute to this evidence, demonstrating very high sensitivity alongside modest specificity, and support the role of POCUS as a feasible adjunct in this clinical context [7–12].

### Strengths and limitations

The study's principal strength lies in its prospective design and implementation in a high-volume, tertiary pediatric emergency setting, enhancing its ecological validity. Moreover, ultrasound evaluations were conducted independently and blinded to clinical outcomes, mitigating the risk of confirmation bias. Physicians achieved high diagnostic performance following limited training, supporting the scalability of this modality in pediatric emergency care.

Nonetheless, certain methodological limitations constrain generalizability. Foremost, the study sample was inherently selective, comprising only patients pre-identified by maxillofacial surgeons as potential candidates for incision and drainage. This introduces spectrum bias, likely augmenting the observed sensitivity and negative predictive value and limiting extrapolation to broader pediatric emergency populations, particularly those presenting with less differentiated or ambiguous clinical features. Additionally, while operator training was standardized, early instructor supervision for the first two scans performed by each operator may have modestly increased diagnostic performance and limited generalizability. In addition, no independent third-party POCUS expert reinterpreted scans in a blinded fashion. Another limitation of our study is the reliance on surgical assessment as the reference standard. Very small volumes (e.g., 0.03 cm^3^) may not be clinically detectable on drainage, potentially leading to underestimation of specificity. Our exploratory use of volume cutoffs suggests that future studies could benefit from predefined quantitative thresholds to complement clinical judgment, thereby providing a more objective basis for determining drainage significance.

### Clinical implications

The findings suggest that POCUS may serve as a valuable diagnostic adjunct in pediatric patients presenting with facial cellulitis of suspected odontogenic origin. Its immediate bedside availability, absence of ionizing radiation, and capacity to inform real-time clinical decision-making render it particularly suited for use in emergency departments importantly, a negative POCUS finding may allow clinicians to safely defer invasive drainage while arranging appropriate dental or maxillofacial follow-up, thereby reducing reliance on radiographic imaging and unnecessary procedures.

### Research implications

To enhance the external validity and clinical adoption of these findings, future studies should employ multi-center designs encompassing more heterogeneous populations, including patients presenting with non-specific facial swelling. Broader inclusion criteria and independent validation of POCUS findings are essential to assess its diagnostic robustness in less selected cohorts. Additionally, further investigations into the integration of POCUS within early diagnostic algorithms, and its downstream effects on clinical outcomes, procedural utilization, and health system resource allocation, will be critical for delineating its role in pediatric emergency practice.

## Conclusion

This study demonstrates the feasibility of POCUS for identifying dentoalveolar abscesses in children presenting with facial cellulitis. The findings suggest that POCUS may help guide management decisions while avoiding unnecessary procedures.

For frontline emergency physicians, incorporating bedside ultrasound into the evaluation of children with facial cellulitis can improve diagnostic confidence, support decision-making, and reduce reliance on radiographic imaging or invasive interventions.

## Data Availability

Data is available following a written request to itaig@hadassah.org.il.

## References

[1] Unkel J, Mckibben D, Fenton MS, Nazif M, Moursi MA, Schuit DK. Comparison of odontogenic and nonodontogenic facial cellulitis in a pediatric hospital population. Pediatr Dent. 1997;19:476–9. 9442541

[2] Ogle OE. Odontogenic infections. Dent Clin North Am. 2017;61(2):235–52. 10.1016/J.CDEN.2016.11.004. 28317564 10.1016/j.cden.2016.11.004

[3] Thikkurissy S, Rawlins JT, Kumar A, Evans E, Casamassimo PS. Rapid treatment reduces hospitalization for pediatric patients with odontogenic-based cellulitis. Am J Emerg Med. 2010;28(6):668–72. 10.1016/J.AJEM.2009.02.028. 20637381 10.1016/j.ajem.2009.02.028

[4] Robertson DP, Keys W, Richardson RR, Burns R, Smith AJ. Management of severe acute dental infections. BMJ. 2015. 10.1136/BMJ.H1300. 25804417 10.1136/bmj.h1300

[5] Miglioretti DL, Johnson E, Williams A, et al. The use of computed tomography in pediatrics and the associated radiation exposure and estimated cancer risk. JAMA Pediatr. 2013;167(8):700–7. 10.1001/JAMAPEDIATRICS.2013.311. 23754213 10.1001/jamapediatrics.2013.311 PMC3936795

[6] Adhikari S, Blaivas M, Lander L. Comparison of bedside ultrasound and panorex radiography in the diagnosis of a dental abscess in the ED. Am J Emerg Med. 2011;29(7):790–5. 10.1016/J.AJEM.2010.03.005. 20825898 10.1016/j.ajem.2010.03.005

[7] Poweski L, Drum M, Reader A, Nusstein J, Beck M, Chaudhry J. Role of ultrasonography in differentiating facial swellings of odontogenic origin. J Endod. 2014;40(4):495–8. 10.1016/J.JOEN.2014.01.002. 24666898 10.1016/j.joen.2014.01.002

[8] Rahmani E, Amani-Beni R, Hekmatnia Y, et al. Diagnostic accuracy of ultrasonography for detection of intussusception in children; a systematic review and meta-analysis. Arch Acad Emerg Med. 2023. 10.22037/AAEM.V11I1.1914. 36919137 10.22037/aaem.v11i1.1914 PMC10008250

[9] Rahmani E, Fayyazishishavan E, Afzalian A, et al. Point-of-care ultrasonography for identification of skin and soft tissue abscess in adult and pediatric patients; a systematic review and meta-analysis. Arch Acad Emerg Med. 2023. 10.22037/AAEM.V11I1.2021. 37609534 10.22037/aaem.v11i1.2021 PMC10440756

[10] Berger T, Garrido F, Green J, Lema PC, Gupta J. Bedside ultrasound performed by novices for the detection of abscess in ED patients with soft tissue infections. Am J Emerg Med. 2012;30(8):1569–73. 10.1016/J.AJEM.2011.08.002. 22030180 10.1016/j.ajem.2011.08.002

[11] Gaspari RJ, Sanseverino A. Ultrasound-guided drainage for pediatric soft tissue abscesses decreases clinical failure rates compared to drainage without ultrasound: a retrospective study. J Ultrasound Med. 2018;37(1):131–6. 10.1002/JUM.14318. 28731535 10.1002/jum.14318

[12] Lam SHF, Sivitz A, Alade K, et al. Comparison of ultrasound guidance vs. clinical assessment alone for management of pediatric skin and soft tissue infections. J Emerg Med. 2018;55(5):693–701. 10.1016/j.jemermed.2018.07.010. 30170835 10.1016/j.jemermed.2018.07.010 PMC6369916

